# Susceptibility Pattern of Caspofungin-Coated Gold Nanoparticles Against Clinically Important *Candida* Species

**DOI:** 10.34172/apb.2021.078

**Published:** 2020-10-18

**Authors:** Zahra Salehi, Azam Fattahi, Ensieh Lotfali, Abdolhassan Kazemi, Ali Shakeri-Zadeh, Saman Ahmad Nasrollahi

**Affiliations:** ^1^Department of Mycology, Pasteur Institute of Iran, Tehran, Iran.; ^2^Centre for Research and Training in Skin Diseases and Leprosy, Tehran University of Medical Sciences, Tehran, Iran.; ^3^Department of Medical Parasitology and Mycology, School of Medicine, Shahid Beheshti University of Medical Sciences, Tehran, Iran.; ^4^Medical Philosophy and History Research Center, Tabriz University of Medical Sciences,Tabriz, Iran.; ^5^Finetech in Medicine Research Center, Iran University of Medical Sciences (IUMS), Tehran, Iran.; ^6^The Russell H. Morgan Department of Radiology and Radiological Science, Division of MR Research, the Johns Hopkins University School of Medicine, Baltimore, MD, USA.

**Keywords:** *Candida albicans*, Non- *albicans Candida*, CAS-AuNPs conjugate, Caspofungin

## Abstract

***Purpose:*** The present study was performed to examine whether caspofungin-coated gold nanoparticles (CAS-AuNPs) may offer the right platform for sensitivity induction in resistant isolates.

***Methods:*** A total of 58 archived *Candida* species were enrolled in the research. The identification of *Candida* spp. was performed using polymerase chain reaction-restriction fragment length polymorphism and *HWP1* gene amplification approaches. The conjugated CAS-AuNPs were synthesized and then characterized using transmission electron microscopy (TEM) and Zetasizer system to determine their morphology, size, and charge. Furthermore, the efficacy was assessed based on the Clinical and Laboratory Standards Institute M60. Finally, the interaction of CAS-AuNPs with *Candida* element was evaluated via scanning electron microscopy (SEM).

***Results:*** According to the TEM results, the synthesized CAS-AuNPs had a spherical shape with an average size of 20 nm. The Zeta potential of CAS-AuNPs was -38.2 mV. Statistical analyses showed that CAS-AuNPs could significantly reduce the minimum inhibitory concentration against *C. albicans* (*P* =0.0005) and non-albicans *Candida* (NAC) species (*P* < 0.0001). All isolates had a MIC value of ≥ 4 µg/ml for CAS, except for *C. glabrata*. The results of SEM analysis confirmed the effects of AuNPs on the cell wall structure of C. globrata with the formation of pores.

***Conclusion:*** According to findings, CAS-AuNPs conjugates had significant antifungal effects against *Candida* spp. Therefore, it can be concluded that the encapsulation of antifungal drugs in combination with NPs not only diminishes side effects but also enhances the effectiveness of the medications.

## Introduction

The growing incidence of invasive candidiasis (IC) has become one of the serious concerns, especially owing to the morbidity and mortality of this infection in immunocompromised individuals.^1^ This is partly due to the limitation of the available antifungals and emergence of resistant isolates to antifungals.^2^ Caspofungin (CAS) is an antifungal agent that induces osmotic instability and yeast apoptosis via targeting beta (1,3)- D-glucan synthase.^3^ Accordingly, it is considered the first-line therapeutic strategy for adult, adolescent, and pediatric patients with IC.^4^ Approximately, over half of candidemia cases receive CAS.^5^

Despite the successful clinical application of CAS, there remains a concern regarding the emergence of drug-resistant species as a result of the extensive use of this antifungal agent.^1^ In general, CAS therapy is a successful approach against most *Candida* spp. However, there are also reports regarding the poor clinical response of this agent to *Candida parapsilosis* and its sibling species, namely *Candida orthopsilosis* and *Candida metapsilosis*.^6,7^

Although the application of metal-drug-conjugates into fungal cells has been efficient in improving the therapy of fungal infections,^8^ they have not been developed at the clinical setting. The MDCs, by combining metals and antifungals in a united structured form, can be promising for the elimination of infections and drug resistance *in vitro*.^8-10^

Gold nanoparticles (AuNPs) are a proper choice in this domain owing to their good physiochemical and safety properties,^11^ ease of synthesis,^12^ and minimal size.^13^ These NPs can be easily combined with various biomolecules, including peptides, enzymes, DNA, and micro molecule medicine.^14^ Accordingly, they can be applied for the induction of drug stability and mitigation of side effects.^15,16^ With this background in mind, the current research was conducted to test the hypothesis stating that CAS-conjugated to AuNPs can provide a proper platform for the sensitivity improvement of the resistant isolates. Regarding this, the aim of the present study was to evaluate the antifungal effects of CAS-AuNPs conjugate and compare these effects with those of CAS and AuNPs as common forms.

## Materials and Methods

### Fungal species

A total of 58 *Candida* spp. isolated from urine, bronchoalveolar lavage, cornea, and blood culture were collected from patients with IC, archived at mycology laboratory at the center for research and training in skin diseases and leprosy, Tehran University of Medical Sciences, Tehran, Iran, were enrolled in the study. All archived isolates were identified as *Candida* spp. using the routine tests. Six out of the 58 strains belonged to *C. parapsilosis* complex identified as *C. parapsilosis* spp. previously.^17^

### Molecular identification of Candida spp. using the polymerase chain reaction-restriction fragment length polymorphism (PCR-RFLP)

DNAs that were extracted from a 24-hour fresh colony, were cultured on Sabouraud Dextrose agar at 37°C (Merck, Germany) according to the isopropanol and the proteinase K method.^18^ The PCR reaction mixture was prepared as follows: 1 μL of DNA, 10 µl of Master Mix RED (Ampliqon, Denmark), and 0.5 µl of each ITS1/ ITS4 primer in a total volume of 25 μL. The PCR program conditions were 95 °C for 10 minutes, 35 cycles of denaturation for 40 seconds at 95 °C, annealing for 40 seconds at 56 °C, an extension for 40 seconds at 72 °C, with the last extension of 8 minutes at 72 °C. The amplicons were analyzed by 1.5% agarose gel electrophoresis in Tris base boric acid EDTA (TBE) buffer (Merck, Germany) using the DNA Safe Stain (Fermentas, USA). The 10 microliters of products were subjected to the* MSP I* restriction enzyme (Fermentas, USA). Restriction fragments were separated by 2% agarose gel electrophoresis.

### PCR amplification of HWP1 Gene

The PCR was performed using the *HWP1* primer *HWP1*-F (5′- GCTACCACTTCAGAATCATCATC-3′) and *HWP1*-R (5′-GCACCTTCAGTCGTAGAGACG-3′) to differentiate the species of *C. albicans* complex. Each mixture contained 12.5 μL of premix 1 μL of DNA template, 0.5 μM of each primer in a total volume of 25 μL. Negative controls were added to each PCR. The reaction mixture was initially denatured at 95°C for 1 minute, followed by 35 cycles of 30 seconds at 95°C, 30 seconds at 60°C, and 1 minute at 72°C, and a terminal extension step of 72°C for 5 minutes. Five microliters of the PCR products were electrophoresed on 1.5% agarose gel in TBE buffer (Merck, Germany), and then observed and photographed under ultraviolet irradiation.

The PCR product was sequenced by the ABI PRISM BigDye Terminator Cycle Sequencing Ready Reaction Kit. The sequences of isolates were subjected to ClustalW pairwise alignment using the MEGA7.0.21 software and edited manually to improve the alignment accuracy and compared in the GenBank database using the BLAST.

### Synthesis of CAS-AuNPs

AuCl_4_ (20 mg) (Sigma- Aldrich, St. Louis, MO, USA) was dissolved in 100 double-distilled water using a magnetic stirrer. Then, 150 mg trisodium citrate (Sigma- Aldrich, St. Louis, MO, USA) was added to the mixture. The temperature of the solution was adjusted to 80°C. The solution mixture was vigorously stirred in this condition until the colour of the solution turned to deep red. The temperature of the solution was cooled down to room temperature. Then, 5 mL of concentrated glycerol was added while being vigorously stirred. CAS (70 mg) (Sigma- Aldrich, St. Louis, MO, USA) was then added to the mixture at room temperature while being vigorously stirred in a magnetic stirrer within 5 minutes. Finally, the mixture was sonicated using a probe-type ultrasonic (400 W, 50% power, 50% cycle) (Bandelin Electronic, Germany) for 10 minutes to form CAS-AuNPs.

### Characterization of CAS-AuNPs

The size and morphology of the synthesized CAS-AuNPs were determined using the transmission electron microscopy (TEM; LEO906- ZEISS, Germany). Furthermore, the hydrodynamic diameter of the synthesized nanoconjugates and polydispersity index were measured by dynamic light scattering (DLS) using the Malvern Zetasizer Nano ZS-90 instrument (Malvern, England). The zeta-potential measurement was also performed by the same instrument.

### Antifungal susceptibility assay

A head-to-head comparison for the efficacy of three compounds including CAS, CAS-AuNPs conjugates, and AuNPs to common* Candida* spp. was performed using the Clinical & Laboratory Standards Institute (CLSI) M- 60.^19^ Briefly, pure powder of CAS was dissolved in sterile dimethyl sulfoxide. The same concentration of CAS-AuNPs was used. The serial dilution of each compound was prepared according to CLSI M-60. The final concentration of each compound was aliquot into 96-well microplates. *Candida* conidial suspension was prepared from 24 hours of fresh culture (1 ×10^6^ cells/ mL). Then, 100 microliters of each suspension were added into the 96-well microplates. The well containing the Roswell Park Memorial Institute (RPMI) 1640 medium (Sigma-Aldrich, St. Louis, MO, USA) and the well containing RPMI 1640- conidial suspension were considered as negative and positive controls, respectively. The minimum inhibitory concentration (MIC) endpoints were evaluated after 24 hours of incubation at 35°C. All experiments were performed in duplicate. The standard strain of *C. parapsilosis* ATCC 22019 was used as quality controls in every run.

The MIC cut-off for CAS was determined based on inhibition of >50% of growth. The non-wild-type/resistant breakpoint for the CAS was as follows for* C. tropicalis*, * C. albicans, C. krusei* CAS ≥1 µg/mL,for 02* C. glabrata* CAS ≥.5 µg/mL*, C. parapsilosis* CAS ≥0.5 µg/mL.

The anidulafungin and micafungin antifungal susceptibilities were conducted to assess whether resistance occurred in the clinical *C. glabrata* isolate. The MIC cut-off for anidulafungin and micafungin was determined based on inhibition of > 50% of growth. The resistant breakpoint for the anidulafungin and micafungin was as follows for *C. glabrata* CAS ≥.5 µg/mLand ≥0.25 µg/mL, respectively.

### Evaluation interaction of CAS-AuNPswith Candida element

Ten microliters of theCAS- AuCl4NPsconjugate at the MIC dose treated with fungal conidia (*Candida glabrata* was chosen as sample) were applied onto a slide surface and left to dry at room temperature. Subsequently, the samples were rinsed several times with Milli-Q water and air-dried again. The dried samples were characterized by Scanning Electron Microscopy (SEM-XL30: Philips, Poland) for morphology.

### Statistical analysis

The statistical analysis was performed by Statistical Package for Social Sciences (SPSS) 22.0 for Windows (SPSS Inc., Chicago, IL, USA).

## Results

### Species identification

**Figure 1:**
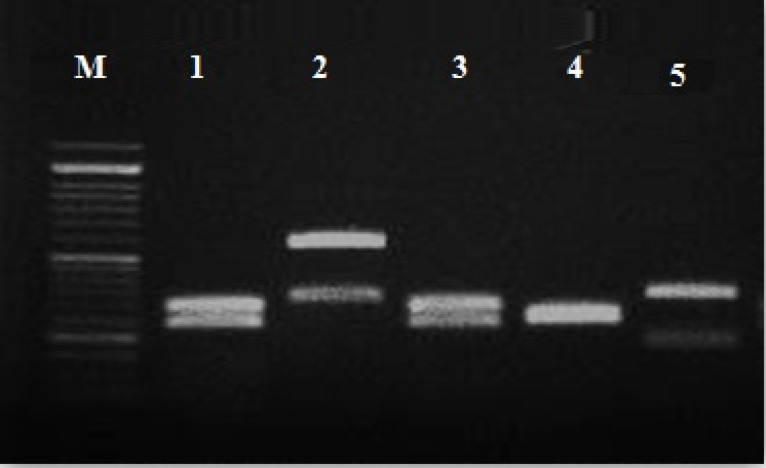
PCR-RFLP gel electrophoresis pattern of *Candida* spp. using the MspI restriction enzyme: M: marker 100 bp; lane 1,3 *C. albicans* (238- 297 bp); lane 2 *C. glabrata* (3.14- 557 bp); lane 4 *C. krusei* (249-261 bp);lane 5 *C. tropicalis* (184- 340 bp).

A total of 52 primary identified *Candida* spp. on CHROM Agar were precisely differentiated using the PCR- RFLP method. Based on RFLP gel electrophoresis *C. tropicalis* (n: 25; ~ 48.07 %)*, C. albicans* (n: 20; ~38.46%),* C. glabrata* (n: 1; 1.92 %)*, C. krusei* (n: 3; ~ 5.76%), were identified (Figure 1).

### PCR amplification HWP1 gene

**Figure 2:**
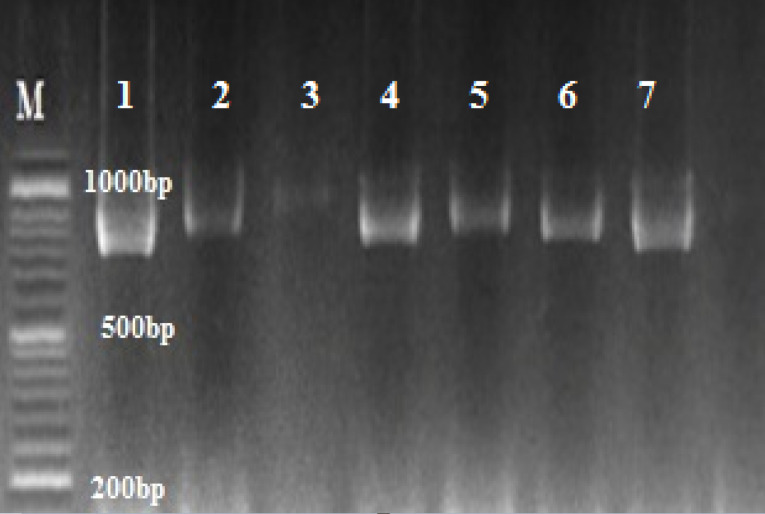
Agarose gel of PCR amplification of *C. albicans* complex with *HWP1* primers; *C. albicans* ~ 900 bp; M: marker 100 bp.

The results of partial amplification of isolates with specific primers of *HWP1* gene yielded fragments of ~900 bp for* C. albicans* (Figure 2). *HWP1* sequences of the isolates were aligned using ClustalW as implemented in MEGA7.0.21 software. The query sequences were paired with those in the NCBI database, using the Blast analysis. All isolates were known as *C. albicans*.

### Characteristics of CAS-AuNPs

**Figure 3:**
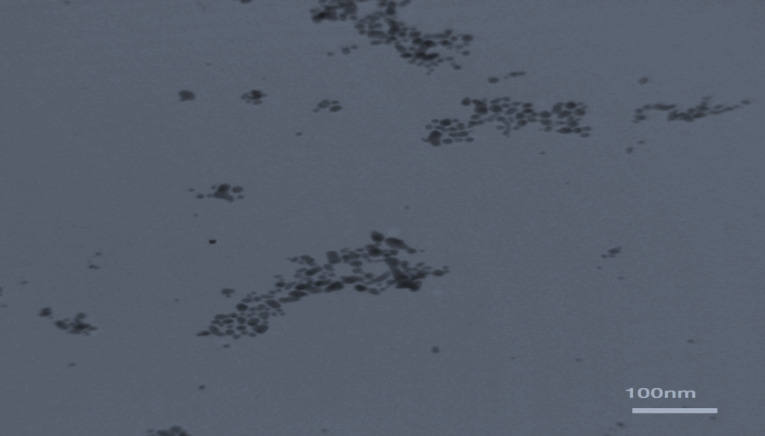
A typical TEM micrograph of the synthesized CAS-AuNPs.

**Figure 4:**
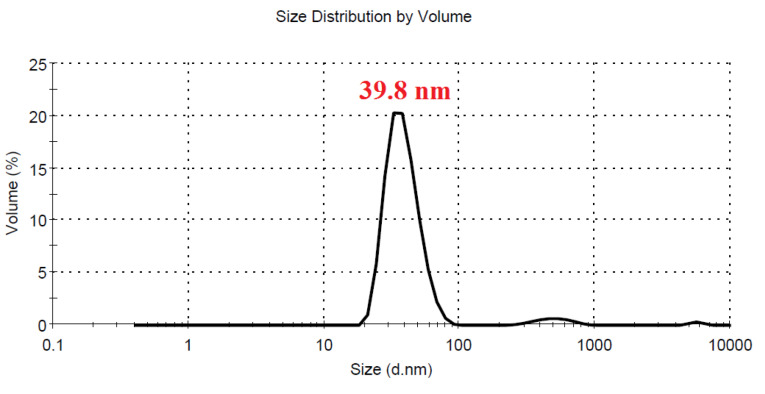
DLS profile of CAS-AuNPs per volume shows the majority of nanoparticles were in hydrodynamic size of 39.8 nm (polydispersity index of 0.395).

**Figure 5:**
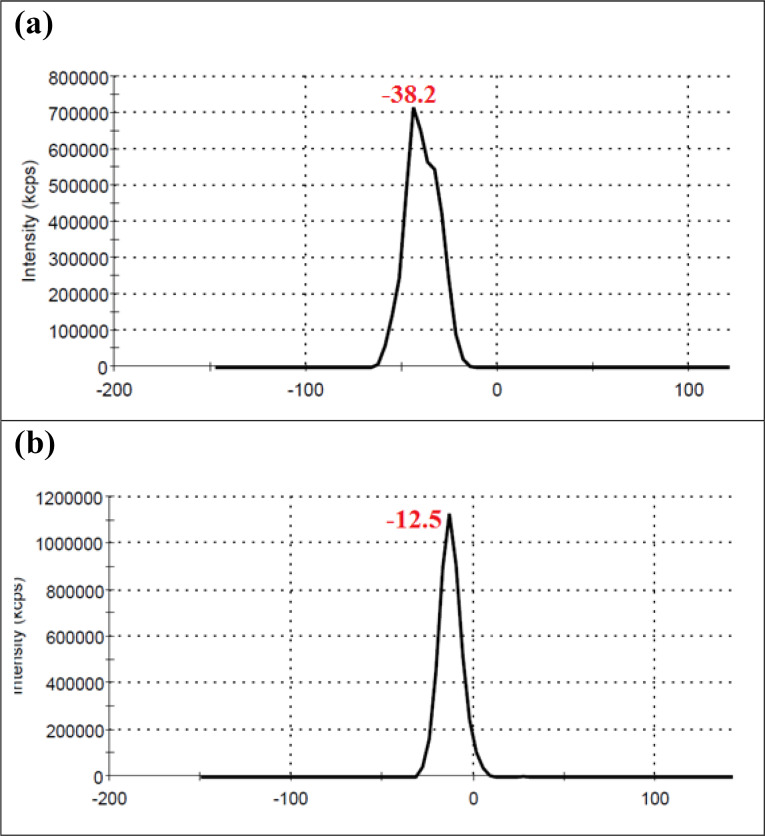
Zeta potential profile of (a) CAS-AuNPs (b) AuNPs.

No significant differences were seen in case of stability of CAS-AuNPs conjugate at 4°C and 25°C, and followed for 6 months when examined by UV- V is spectroscopy. As stated earlier, the CAS-AuNPs conjugate was characterized using the TEM) and Zeta-sizer to determine the morphology, size, and charge of nano conjugates. According to TEM images, the synthesized CAS-AuNPs were observed in a spherical shape with an average size of the nanoparticles ~ 20 nm (Figure 3). The hydrodynamic size of nano conjugates (per volume) ranged from 30 to 50 nm and the polydispersity index was 0.395 (Figure 4). The Zeta potential of CAS-AuNPs was obtained as -38.2 mV (Figure 5), demonstrating that the synthesized nano conjugate exhibited good stability. It is well-known that one of the most common applications of Zeta potential data is to show the colloid stability of a synthesized nanoconjugate, as the guidelines classify the values of ± 0–10 mV, ± 10–20 mV and ± 20–30 mV and ˃ ± 30 mV as the evidence of highly unstable, relatively stable, moderately stable and highly stable nanoconjugates.^20^

Moreover, by comparing the Zeta potentials of AuNPs and CAS-AuNPs (Figure 5a & b), it may be understandable that caspofungin was attached to the surface of AuNPs. As shown in Figure 5, the zeta potential of AuNP was -12.5 mV and shifted to -38.2 mV after conjugation with CAS molecules that resulted from the presence of charged functional groups in the structure of CAS.^20,21^

### Results of antifungal susceptibility assay

The MIC_50_ for CAS and CAS-AuNPs conjugate was 0.125 µg/mL and 0.125 µg/mL compared to 0.03 µg/mL and 0.06 µg/mL for *C. albicans*, *C. tropicalis*, respectively.

The MIC ranges for CAS and CAS-AuNPs conjugate were 0.12- 0.5 µg/mL, 0.25 µg/mL, and 4 µg/mL compared to 0.06- 0.12 µg/mL 0.06-0.25 µg/mL, and 0.06 µg/mL for *C. parapsilosis*, *C. krusei*, and * C. glabrata,* respectively. Statistical analyses showed that CAS-AuNPs could significantly reduce the MIC against *C. albicans* (*P*= 0.0005) andnon*-albicans Candida* (NAC) (*P* < 0.0001). The results revealed that the degrees of the susceptibility of *Candida* spp. was increased to CAS-AuNPs conjugate. Among all isolates, the MIC of ≥4 µg/mL for CAS was observed except for *C. glabrata*. According to the MIC of anidulafungin and micafungin for *C. glabrata*, the mentioned isolate was defined as CAS resistance isolate (Table 1). This species showed resistance to fluconazole (MIC:16) and itraconazole (MIC=8) *in vitro* and clinicpreviously.

**Table 1:** *In-vitro* susceptibility of clinical isolate of *C. glabrata* to echinocandin

**Echinocandin antifungal**	**MIC (µg/mL)**
Anidulafungin	≥ 2
Micafungin	≥ 1
Caspofungin	4≤

MIC: minimal inhibitory concentration.

**Figure 6:**
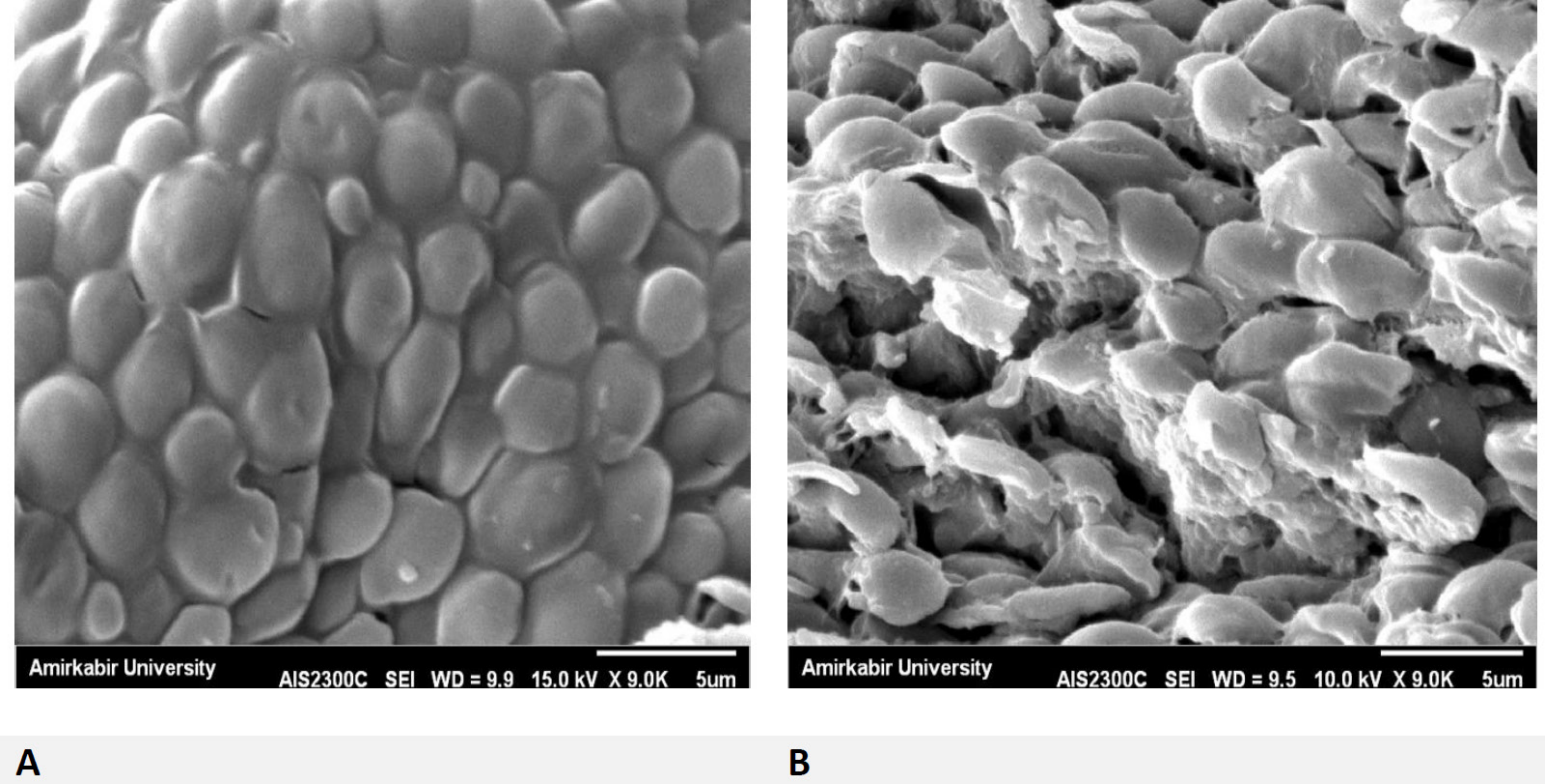
SEM image of resistant *C. glabrata* strain. Comparison of normal cells (A) and cells were damaged (B) *C. glabrata* cellsin exposed to 0.06 µg/mL concentration of CAS-AuNPs (10 ppm).

Regarding this observation, we found CAS conjugated with AuNPs have synergistic effects, because of their low MIC ranges. Thus, they have more potency than AuNPs-single against CAS-resistant *C. glabrata*. Figure 6 indicates membrane damage as well as cell wall plus cell death in yeast cells’ exposure to CAS-AuNPs conjugated compared to normal yeast cells with a healthy cell membrane. Accordingly, significant effects of antifungal drugs conjugated with gold nanoparticles provide new solutions for the treatment of invasive fungal infections.

## Discussion

CAS is known as a safe and effective agent in the treatment of IC. Meanwhile, NPs are considered desirable for drug delivery against fungal infections due to their small and controllable size, as well as larger surface area.^22^ The present study was the first attempt toward synthesizing CAS-AuNPs conjugate and investigating its effect against *Candida* spp.

The antifungal property of NPs depends on their synthesis method, as well as their concentration and size. In this regard, a smaller size results in greater antifungal susceptibility. In the present study, the results of TEM and DLS revealed that AuNPs had a spherical particle shape. Furthermore, they had a PDI of < 0.5 with a Zeta potential of > ±15, indicating good stability for the synthesized nano-conjugate. With regard to CAS-AuNPs, they showed a particle size of 10 nm with an antifungal activity of 0.06-0.5 µg/mL. The same result was reported in a study using AuNPs-conjugated fluconazole against the fluconazole-resistant strains of *C. albicans*, where the nano-conjugate had a size of 10 nm with an antifungal activity of 2 µg/mL.^22^

In a study performed by Abd et al, the size of zinc oxide (ZnO) NPs was calculated as 50 nm, which showed an antifungal activity of 5.8 µg/mL.^23^ Furthermore, in a recent study, Hosseini *et al*. reported that ZnO NPs were spherical with a diameter size of 20-40 and an antifungal activity of 0.02-18.1 µg/mL.^24^ In the current study, the nano-conjugate had a spherical shape with the size range of 7-15 nm and antifungal activity of 0.06 µg/mL. Our results showed that CAS-AuNPs conjugate was more effective than single CAS or AuNPs against *Candida* spp. Among 58 *Candida* strains, there was only one resistant isolate (*C. glabrata*). On the other hand, all isolates were sensitive to CAS-AuNPs conjugate. According to the MIC results, it can be stated that the NPs have an intensive antifungal property; therefore, they are a suitable choice for drug-resistant isolates.

In the present study, the reduced growth of the standard strain *C. parapsilosis* ATCC 22019 confirmed the potential antifungal activity of CAS-AuNPs against both *C. albicans* and NAC isolates. In line with the present research, some studies have also discussed the potential antifungal power of NPs. In this regard, silver (Ag) NPs have been used as a new generation of antifungal agents in recent years.^25^ Gajbhiye et al observed the maximum antifungal activity of Ag-NPs conjugated with fluconazole against *C. albicans*, followed by *Phoma glomerata*.^26^ Furthermore, Monteiro et al^27^ demonstrated the antifungal and antibiofilm activities of nystatin-conjugated Ag-NPs against *Candida* spp.

Zawrah *et al* reported the antifungal activity of fluconazole coated with AuNPs against *Aspergillus niger*, *A. flavus,* and *C. albicans* using the agar disk diffusion method.^28^ In a study, Memarian et al investigated a *C. albicans* resistant to fluconazole and reported that this strain was sensitive to fluconazole-AuNPs conjugate,^22^ which is in line with our results. In addition, Sarrafha et al showed the high activity of nano-liposomes containing fluconazole against *A. flavus* and *A. fumigatus*.^29^ The use of fluconazole has been limited nowadays due to the prevalence of fungal resistance.^30^ In this study, CAS-AuNPs conjugate was synthesized for the first time. It is suggested to investigate the antifungal activity of this compound against different fungal strains.

As our results indicated, CAS-AuNPs had high levels of inhibitory activity against echinocandin-resistant* C. glabrata*. It can be assumed that the emerged resistance to one agent can be managed by the use of AuNPs, which facilitates susceptibility enhancement. The results of SEM analysis confirmed the effects of AuNPs on the membrane and cell wall structure of the *C. glabrata* exposed to CAS-AuNPs, which induced the formation of pores on the cell wall and finally cell death.

Overall, the CAS-AuNPs conjugates showed significant antifungal effects on *Candida* spp. through the destruction of the membrane and cell wall integrity. Accordingly, it can be concluded that the encapsulation of antifungal drugs in combination with NPs leads to the reduction of side effects and enhancement of medication effectiveness. However, the confirmation of this finding requires further investigation of comparative data from well-controlled trials and clinical studies evaluating the safety of CAS-ANPs conjugate at clinical settings.

## Conclusion

The CAS-AuNPs conjugates showed significant antifungal effects on *Candida* spp. through destruction of membrane and cell wall integrity. Note that the use of encapsulation of antifungal drugs in combination with NPs leads to diminished side effects and enhanced effectiveness of the drugs. However, confirmation of this finding needs further investigation on comparative data from well-controlled trials as well as clinical experience including evaluating the safety of CAS-AuNPs conjugate in clinical settings.

## Conflict of Interest

The authors declare that they have no conflict of interest.

## Ethical Issues

Not applicable.
